# New protocol for kinetic assay seeding ability recovery “KASAR” from formalin-fixed paraffin-embedded tissues

**DOI:** 10.3389/fmolb.2023.1087982

**Published:** 2023-01-30

**Authors:** Monica Hepker, Griffin Clabaugh, Huajun Jin, Anumantha G. Kanthasamy

**Affiliations:** ^1^ Parkinson Disorders Research Laboratory, Iowa Center for Advanced Neurotoxicology, Department of Biomedical Sciences, Iowa State University, Ames, IA, United States; ^2^ Center for Neurological Disease Research, Department of Physiology and Pharmacology, University of GA, Athens, GA, United States

**Keywords:** biomarker, RT-QuIC, formalin-fixed, kinetic assay, alpha-synuclein, protein aggregation, Parkinson's disease, FFPE

## Abstract

The real-time quaking-induced conversion (RT-QuIC) alpha-synuclein (aSyn) protein kinetic seeding assay has been very useful for detecting pathological aggregates in various synucleinopathies including Parkinson’s disease (PD). This biomarker assay relies on fresh frozen tissue to effectively seed and amplify aSyn aggregating protein. With vast repositories of formalin-fixed paraffin-embedded (FFPE) tissues, it is paramount to harness the power of kinetic assays to unlock the diagnostic potential of archived FFPE biospecimens. However, the major challenge posed by significantly reduced amplification of formalin-fixed tissues in the assay suggests that formalin fixation deterred monomer interaction with the sample seed and depressed subsequent protein aggregation. To overcome this challenge, we developed a kinetic assay seeding ability recovery (KASAR) protocol to maintain the integrity of the tissue and seeding protein. For this, we implemented a series of heating steps with the brain tissue suspended in a buffer composed of 500 mM tris-HCl (pH 7.5) and 0.02% SDS after the standard deparaffinization of the tissue sections. Initially, samples from seven human brain samples, including four samples from patients diagnosed with dementia with Lewy bodies (DLB) and three samples from healthy controls without DLB, were compared to fresh frozen samples under three different, but clinically common sample storage conditions: formalin-fixed, FFPE, and FFPE slices cut 5 µm thick. The KASAR protocol was able to recover seeding activity for all positive samples in all storage conditions. Next, 28 FFPE samples from the submandibular gland (SMG) of patients diagnosed with PD, incidental Lewy body disease (ILBD), or healthy controls were tested with 93% of results replicating when blinded. With samples of only a few milligrams, this protocol recovered the same quality of seeding in formalin-fixed tissue as fresh frozen tissue. Moving forward, protein aggregate kinetic assays, in conjunction with the KASAR protocol, can be used to understand and diagnose neurodegenerative diseases more comprehensively. Overall, our KASAR protocol unlocks and restores the seeding ability of formalin-fixed paraffin-embedded tissues for the amplification of biomarker protein aggregates in kinetic assays.

## 1 Introduction

RT-QuIC is a protein kinetics assay that has been widely used to study the seeding activity of infectious prion proteins and many other disease-associated proteins that show evidence of aggregation. It is currently being employed to screen various tissues in human patients for the biomarker aggregated alpha-synuclein (aSyn). A great deal of interest has been expressed in using RT-QuIC in conjunction with other diagnostics to help detect Parkinson’s disease (PD) and other synucleinopathies in their early stages when intervention can have the greatest impact on preserving or extending the quality of life.

In human medical practice, tissue samples from many areas of the body are commonly collected for histologic evaluation by a pathologist. Typically, these tissue samples will be preserved in a commercial preparation of 10% neutral-buffered formalin (NBF) solution for at least 24 h before being embedded in paraffin wax. Once formalin-fixed and paraffin-embedded (FFPE), tissues and the associated protein biomarkers are stable at room temperature for several years, even decades. Despite some of the formalin-induced bonds being spontaneously reversible, most of them are not (Eltoum et al., 2001). Formalin has been known for centuries to almost permanently preserve tissues in a state of near perfection. First, the small, highly reactive formaldehyde molecule easily penetrates membranes. Then, in a series of condensation reactions, formaldehyde creates, among other products, methylene bridges with a variety of peptides, proteins, and other cellular components (Rourke and Padula, 2016). These methylene bridges are believed to be the cornerstone of formalin’s success as a fixative, and breaking them is the key challenge to overcome for recovering the sample proteins’ assay seeding potential.

For the last several decades, histopathological evaluation of samples has taken advantage of a technique for identifying specific antigens using immunohistochemistry (IHC). In this technique, thin slices of FFPE tissues are mounted on slides, deparaffinized, and then rehydrated in a series of xylene, alcohol, and PBS washes. Once the fixed tissue is rehydrated, highly specific monoclonal antibodies can be used to detect biomarker proteins. If the protein antigens are inaccessible to the specific antibodies, they can often be uncovered by additional processing. One common technique, called heat-induced antigen retrieval (HIAR), utilizes high heat to weaken and break the covalent bonds created by formalin, thereby allowing the antibodies employed by IHC to bind with their antigen epitope (Rourke and Padula, 2016). Recently, genomic and proteomic studies have sought ways to break the covalent bonds of formalin fixation and free the genetic material (Al-Attas et al., 2016; Berrino et al., 2020; Frazer et al., 2020; Panchal et al., 2020; Nguyen et al., 2022) or cellular proteins (Kawashima et al., 2014; Lai et al., 2016; Mason, 2016; Rourke and Padula, 2016; Coscia et al., 2020; Griesser et al., 2020; García-Vence et al., 2021; Pöschel et al., 2021; Thacker et al., 2021) for analysis, involving prolonged incubation at high heat or enzymatic digestion. To unlock the diagnostic potential of vast repositories of FFPE tissues, it is paramount to harness the power of kinetic assays. However, until now, researchers using protein aggregation kinetic assays have not adopted techniques for routinely utilizing FFPE samples, despite their significant implications for neurobiochemical and diagnostic assays.

In the present study, to run RT-QuIC assays on FFPE samples, we applied the principles from HIAR (heat-induced antigen retrieval and the use of tris and heat for bond-breaking), resulting in the kinetic assay seeding ability recovery (KASAR) protocol. We show that by treating FFPE samples with the KASAR protocol, a significant recovery of assay seeding activity is achieved. This opens the potential to find ultra-low amounts of specific biomarkers in samples that are years or even decades old. The KASAR protocol is based on several well-established methods of processing a biological sample to ultimately restore assay seeding activity (Sternbach et al., 1997; Kawashima et al., 2014; Lai et al., 2016; Fenyi et al., 2021; Kapoor et al., 2021). Thus, by understanding the basic biochemistry underlying those methods and the protein aggregate, it is possible to identify the best combination of techniques to create a suitable KASAR protocol for a given sample.

## 2 Materials and methods

### 2.1 Patient samples

#### 2.1.1 Brain

Fresh frozen blocks of human brain globus pallidus (GP) tissues were obtained from UC Davis and stored at -80°C until use. Postmortem diagnosis confirmed that the tissues represent 4 dementia with Lewy bodies (DLB) and 3 healthy control individuals.

Human brain samples were cut from the GP tissue blocks stored at -80°C. Each sample was cut on an ice-cold, sterile surface with a new razor blade to avoid the risk of cross-contamination. Approximately 30–50 mg of each sample was weighed and processed immediately. Sample tissue and sufficient PBS to create a 10% w/v solution were placed in 1.5-ml screw-cap RINO tubes along with approximately 100 mg zirconium oxide beads (OPS Diagnostics) and mixed in a Bullet Blender for 5 min at maximum speed. The resulting homogenates were centrifuged at 10,000 x g for 5 min to reduce foam, then pulse vortexed briefly at high speed. The resuspended 10% homogenate was transferred into 1.5-ml low protein-binding tubes and stored at -80°C. Residual beads and tubes were also stored at -80°C. For RT-QuIC, fresh frozen homogenate was thawed and diluted in PBS then 2 μl per well was applied to the plate.

#### 2.1.2 Formalin-fixed brain

An additional 50–70 mg piece of tissue from each frozen GP tissue block was placed in fine-mesh histology cassettes and submerged in 10% NBF for at least 18 h. Approximately 20 mg was then homogenized in the same manner as fresh frozen tissue to create formalin-fixed sample homogenates.

#### 2.1.3 Formalin-fixed paraffin-embedded brain

The remaining GP pieces from fixed DLB and control brains were sent to ISU Veterinary Pathology Department for processing and embedding using industry-standard protocols. Samples were prepared to simulate a needle biopsy sample. Tissue was cut and embedded such that the surface area at the face of the block would be approximately 5–15 mm^2^.

Of the paraffin-embedded DLB and control samples, a small subsample was cut directly from the embedded block and processed the same as a “core sample”, prior to cutting and slide-mounting a set of slide-mounted FFPE sections.

We scraped 4- to 6-µm thick, slide-mounted sections into 1.5-ml low-binding tubes using #9 RD steel razor blades (0.009”). Small drops of ethanol were pipetted onto each slide prior to scraping to help reduce static charge effects. Additionally, 6 serial 4-µm thick sections were cut and immediately placed in a single 1.5-ml Eppendorf tube.

#### 2.1.4 Submandibular gland

Slide-mounted FFPE SMG tissue sections were provided by the Banner Sun Health Research Institute (BSHRI; Phoenix, AZ). SMG FFPE tissues from 13 PD, 3 incidental Lewy body disease (ILBD), and 12 healthy control subjects were represented. All persons were recruited and clinically followed to autopsy and neuropathological examination as part of the Arizona Study of Aging and Neurodegenerative Disorders/Brain and Body Donation Program (www.brainandbodydonationprogram.org). Thus, a separate Institutional Review Board (IRB) approval from Iowa State University was not required. All cases were neuropathologically examined using specific diagnostic criteria for PD based on previously published criteria (Manne et al., 2019). Samples with Lewy bodies that did not meet the diagnostic criteria for PD were classified as ILBD. Controls consisted of normal elderly subjects or subjects with AD-related changes but insufficient evidence for AD diagnosis.

#### 2.1.5 Formalin-fixed paraffin-embedded SMG slides

We scraped 5-µm thick FFPE SMG sections into 1.5-ml low-binding tubes using #9 RD steel razor blades. Small drops of ethanol were also pipetted onto each slide prior to scraping to help reduce static charge effects.

### 2.2 RT-QuIC

The RT-QuIC assay was generally performed as previously described (Manne et al., 2020) using a 96-well clear-bottom plate (Nalgene Nunc International) with each well loaded with 5–7 800-µm silica beads (OPS Diagnostics). For all RT-QuIC assays, the reaction mixture consisted of final concentrations of 40 mM phosphate buffer (pH 8.0), 170 mM NaCl, 10 μM ThT, 0.00125% sodium dodecyl sulfate (SDS) and 0.1 mg/ml of recombinant aSyn filtered through a 100-kDa cutoff filter. Each reaction consisted of 2 µl of test sample as seed and 98 µl of aSyn RT-QuIC reaction mixture per well of a 96-well plate preloaded with six 0.8-mm diameter silica beads (OPS Diagnostics). Next, plates were covered with clear sealing film and run on a BMG CLARIOstar. Plate runs involved alternating 1-min 400-rpm double orbital shaking periods with 1-min rest periods, incubation at 42°C, and fluorescent readings every 45 min. The assay run time was 24 h for brain, and 36 h for SMG.

### 2.3 Bradford protein estimation

Protein estimation was conducted using the Bio-Rad Protein Assay Dye Reagent Concentrate (Cat. #5000006) in clear Nunc 96-well plates with bovine serum albumin (BSA) standard from 0 to 14 μg/ml.

### 2.4 Kinetic assay seeding ability recovery (KASAR) protocol

If the sample is embedded in paraffin, steps to deparaffinize must be done prior to KASAR treatment. If the tissue is simply formalin-fixed or has already been deparaffinized, the recovery protocol can be started directly.

#### 2.4.1 Deparaffinization

We added 950 µl of xylene to FFPE tissues in 1.5-ml tubes and then vortexed using a rotary mixer for 5 min (Intelli-Mixer, setting U2, 95). Samples were incubated at 56°C for 3 min at 300 rpm on a ThermoMixer to melt the paraffin followed by centrifugation at 25°C for 5 min (13,000 x g). The supernatant was removed using a pipette while being careful not to disturb the pellet, thus some xylene remaining at the bottom with the pellet was acceptable. Next 950 µl of 100% ethanol was added to each tube. Samples were mixed for 5 min (Intelli-Mixer, setting U2, 95) followed by centrifugation at 25°C for 5 min (13,000 x g). The supernatant was removed without disturbing the pellet. The ethanol wash was repeated, and the supernatant was removed. To remove the remaining ethanol, we used a SpeedVac (Savant SPD1030, Thermo Scientific) on its lowest setting for 10 min. Alternatively, tubes may be left open at room temperature until the ethanol has completely evaporated leaving a visibly dry pellet of deparaffinized tissue.

#### 2.4.2 KASAR

Once the pellets of deparaffinized or formalin-fixed tissue were visibly dry, 10–15 0.4-mm zirconium (OPS) grinding beads were added to each sample. Then, 950 µl of 500 mM tris + 0.02% SDS buffer (TSB, pH 8.0) was added and mixed for 1 min (Intelli-Mixer, setting U2, 95). To further break up the pellet, the samples went through 3 freeze-thaw cycles. A −80°C ethanol bath was used to flash-freeze the sample and a warm-water bath was used to quickly thaw the tissue. Each bath immersion was about 5 min to ensure completion of each freeze-thaw cycle. The samples were again mixed for 10 min (Intelli-Mixer, setting U2, 95) followed by centrifugation at 25°C for 5 min (13,000 x g). The supernatant was removed by pipette without disturbing the pellet. Fresh 1 x TSB was then added and mixed for 10 min (Intelli-Mixer, setting U2, 95). Samples were centrifuged at 25°C for 5 min again (13,000 x g). The supernatant was removed by pipette to retain 90 µl (SMG) or 15 µl (Brain) TSB per slide in each tube (e.g., if 6 slides were scraped into the tube, 6 × 15 µl = 90 µl needs to be retained, thus 950 μl—90 µl = 860 µl of supernatant was removed). Following supernatant removal, the samples were placed in the ThermoMixer at 1500 rpm and 90°C for 20 min. The temperature was reduced to 60°C for an additional 90 min and continued 1500-rpm shaking. Next, samples were mixed (Intelli-Mixer, setting U2, 95) for 5 min following the incubation. Finally, samples were sonicated for 8 min at 90% with cycles of 30-s intervals of sonication and 30 s of rest for a total sonication time of 4 min.

### 2.5 Statistical analysis

GraphPad 7.0 was used for statistical analysis. The number of biological replicates is expressed as “n” unless otherwise mentioned. Raw data were normalized as a percentage of the maximum relative fluorescence unit (% Max RFU) to facilitate data comparison across plates that were all run with the gain on the plate reader set to a level that avoids oversaturating the asymptotic phase of the aggregation curve. To calculate the % Max RFU, the mean of 3 technical replicates per sample was found for all time points (45-min intervals of 24- or 36-h run times). Readings that over-saturated the plate reading were excluded as the gain is set to permit most readings at 25% of the maximum detectible. Next, the highest mean RFU per run time for each sample is then taken as a percentage of the highest or Max RFU on that plate. A reaction was considered positive when two or more wells crossed the aggregation threshold, which was calculated as the average background fluorescence plus five standard deviations, or approximately 10%, before the run time was complete. This calculation selects for and compares sample performance during the assay run.

## 3 Results

### 3.1 αSyn RT-QuIC seeding activity of fresh frozen vs. formalin-fixed brain tissues

In the first experiment, to assess the seeding ability of formalin-fixed material compared to fresh material, we compared fresh frozen and formalin-fixed brain tissue homogenates from 4 DLB patients and 3 healthy control subjects. Each sample was cut, with one-half fixed in 10% NBF followed by processing into 10% w/v homogenates and the other half of the frozen tissue directly processed into 10% w/v homogenates. The resulting homogenates were diluted to 0.01% (10^−4^ fold dilution), then run on RT-QuIC. A Bradford protein estimation was conducted on fresh frozen tissues using 10% homogenate samples contained approximately 3–7 μg/μl of total protein. Adding 2 μl of those samples when diluted to 10^−4^ resulted in 6–14 ng of sample protein loaded per well in the RT-QuIC assay.

**FIGURE 1:**
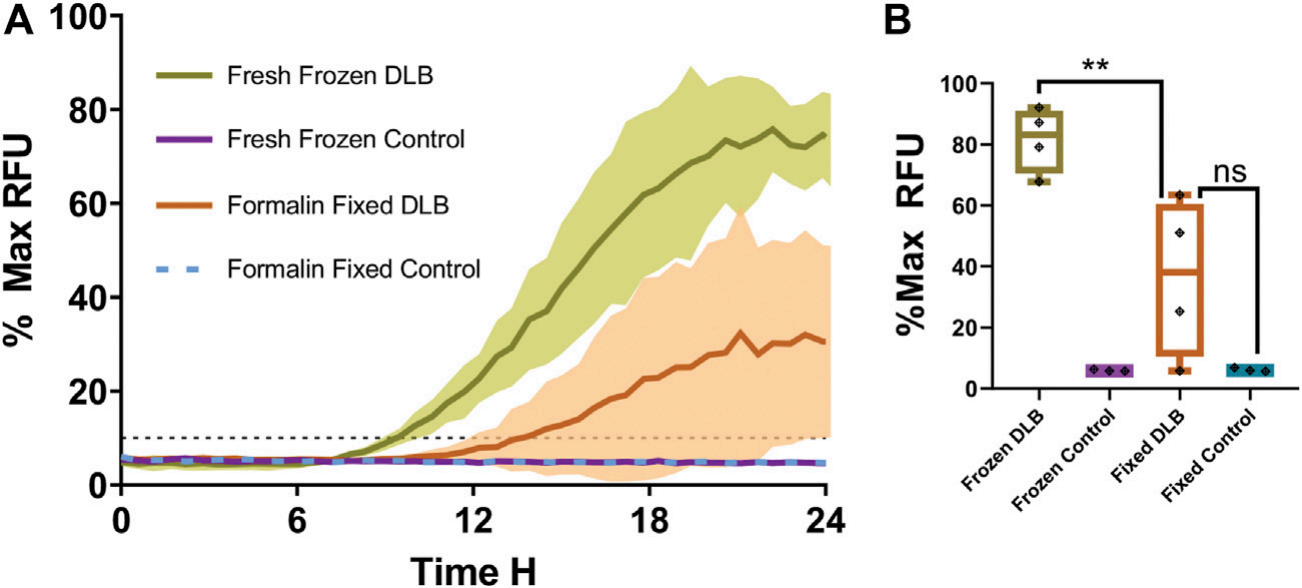
Fresh frozen versus formalin-fixed brain tissue in RT-QuIC. **(A)** The overall mean fluorescence of fresh DLB positive brain homogenate (green) vs. formalin-fixed DLB positive brain homogenate (orange) and controls (teal and violet) as a percentage of the peak fluorescence (% Max) reading. The shaded areas indicate the standard deviation of the readings over time. The black dotted line indicates the calculated aggregation threshold above which indicates positive (n = 4) (fluoresces strongly) whereas failing to cross the threshold indicates negative (n = 3) readings. **(B)** % Max RFU of the mean of each sample. Results were analyzed using t-test. Data expressed as the mean of three technical replicates. ** = *p*< 0.01 and not significant (ns) when *p* > 0.05.

The results were grouped by treatment to determine the effect of formalin fixation on DLB positive and control samples. DLB (n = 4) and normal controls (n = 3) were compared. Each sample was run in triplicate. The fresh frozen samples of each specimen were also run in triplicate. The mean of wells seeded with fresh frozen samples from 4 DLB patients started to amplify at approximately 8 h, and their RFU peaked near 80% Max RFU, while the mean of 3 fresh frozen control samples did not amplify during the run. The standard deviation of the results is shaded and bordered, with fresh frozen DLB samples in olive green, and formalin-fixed DLB samples in orange (Figure 1A). The controls of both fixed and fresh frozen groups failed to amplify which is typical for fresh samples and expected in fixed control samples (Figure 1A). Amplification of formalin-fixed tissues from DLB patients, represented in orange, was delayed with significant depression in intensity, the mean of all samples reaching only 35% Max RFU. Also, 50% of formalin fixed DLB samples failed to have 2 or more wells cross the threshold. No formalin-fixed control samples amplified during the run. When comparing the Max RFU of each sample relative to the strongest amplifying sample (Figure 1B), the peak reading (% Max RFU) of formalin-fixed DLB samples was significantly (*p* = 0.0087) less than the unfixed DLB samples. In contrast, fixed DLB samples and the fixed control samples did not differ significantly (*p* = 0.1058), which means these groups cannot be separated from each other if run in a blinded fashion. These results indicate that the process of formalin fixation alone has a significant impact on sample seeding ability.

### 3.2 KASAR treatment recovers seeding activity in FFPE core brain tissue

**FIGURE 2:**
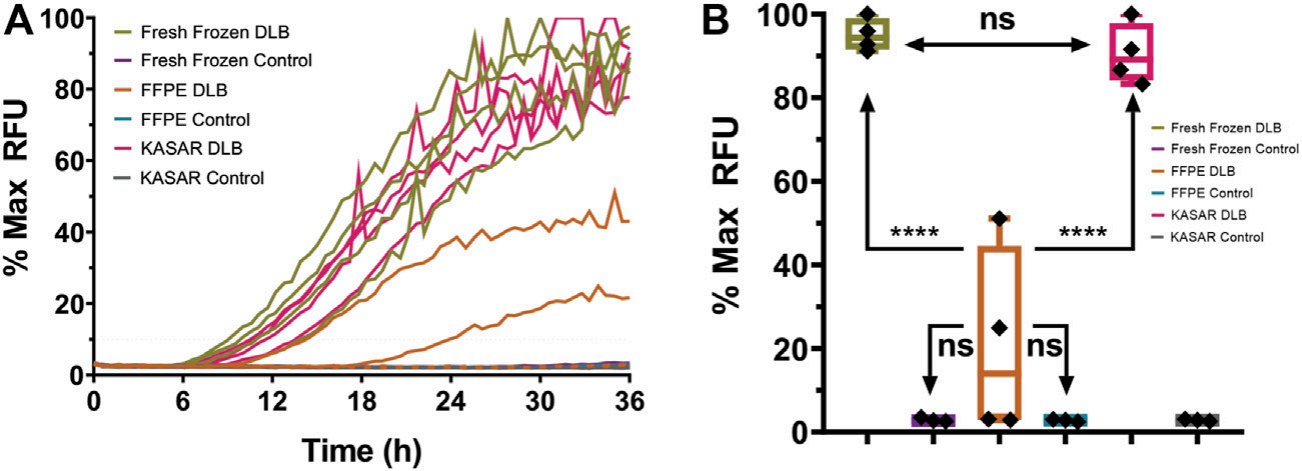
RT-QuIC results for fresh frozen, FFPE Core, and KASAR-treated FFPE core brain tissues. **(A)** Mean amplification curves of four individual fresh frozen samples of DLB (green with open circles) and three control (purple with black triangles) brain homogenates run in triplicate, the same DLB (orange with x’s) and control (blue with open triangles) brains after processing and being embedded into wax blocks, and the same DLB (magenta) and control (gray) brains again after KASAR treatment. The graphs of all KASAR-processed samples are nearly indistinguishable from the fresh frozen results. **(B)** Quantitative analysis of the mean % Max RFU showing that the seeding activity of KASAR-treated FFPE “core” samples was nearly fully recovered relative to fresh frozen samples. Two out of four FFPE DLB samples were false negatives and failed to cross the aggregation threshold determined by fluorescence greater than 10% Max RFU. The other two FFPE DLB samples did cross the threshold but have a reduced % Max RFU compared to fresh and KASAR-treated FFPE samples. Results were analyzed using t-test. Data expressed as the mean of three technical replicates. Ns, not significant.

To further determine whether our KASAR protocol recovers the seeding activities in FFPE “core” brain samples weighing several milligrams, four DLB and three control FFPE “core” brain samples were processed, which were represented by 10- to 20-mg samples of FFPE brain tissue removed from the block, deparaffinized and processed into 10% w/v homogenates. The results indicate that a relatively large sample weighing several milligrams, like when a sample core is removed from an FFPE block using a biopsy punch, can be successfully processed using the KASAR method. Both the fresh frozen and FFPE “core” brain samples were used to seed an RT-QuIC assay (Figure 2A). Formalin-fixed and FFPE tissues reacted similarly in the assay. Each % Max RFU was depressed and half of the DLB samples failed to cross the threshold, a false negative result for the biomarker, compared to the fresh tissue homogenate where all DLB samples amplified. Formalin fixation causes even highly reactive samples to amplify weakly or not at all in the assay. Beyond fixation, the process of embedding and rehydrating tissues did not significantly alter sample performance in the assay.

FFPE core samples were then subjected to a modified KASAR protocol. Briefly, the surface area of deparaffinized core samples was increased by homogenization in PBS using a bullet blender. After this coarse grind, the PBS supernatant was removed and a volume of TSB equal to twice the PBS for the 10% homogenate was added to the grinding beads and residual FFPE sample. The tris-suspended samples were then heated and sonicated according to the KASAR protocol described in the Materials and Methods. A Bradford protein estimation assay was performed to estimate the approximate amount of total protein present after processing, and the samples were diluted to approximately 0.01–0.05 μg/μl for an RT-QuIC assay. Not only was the seeding activity of FFPE core DLB samples significantly improved by the KASAR treatment, but also these samples were no longer statistically distinguishable from fresh frozen DLB tissues (Figure 2B). In contrast, FFPE DLB samples not processed with the KASAR protocol were indistinguishable from both fresh and fixed control samples.

### 3.3 Whole-sectioned SMG FFPE slides processed with KASAR in blinded RT-QuIC

To determine if the KASAR protocol was effective in other tissue types, 28 SMG FFPE samples on slides from PD, ILBD, and normal controls were evaluated. The samples were deparaffinized and processed with the KASAR protocol with 2 µl of undiluted sample being added to each well. KASAR-treated SMG FFPE slide samples subjected to RT-QuIC generated results similar to previously published results of fresh frozen tissue where samples from patients with PD amplified and control samples failed to amplify (Manne et al., 2020).

**FIGURE 3:**
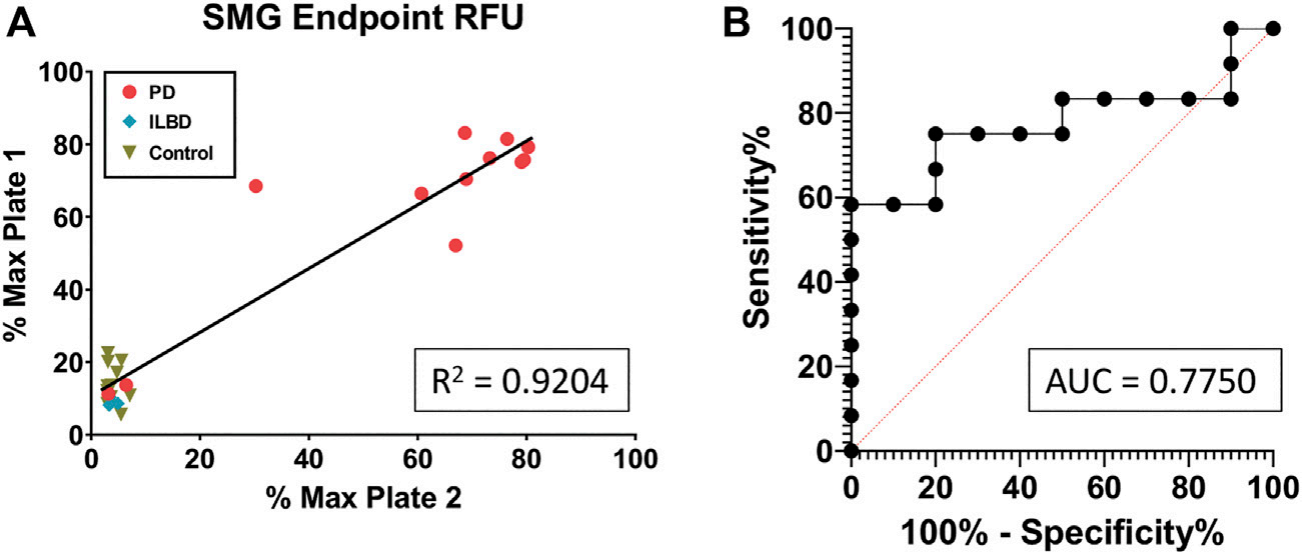
KASAR-treated FFPE SMG slide-mounted samples demonstrate repeatability across runs. **(A)** Simple linear regression of % Max RFU of (n = 28) samples run *via* RT-QuIC between two different plates, using an unblinded run as the predictor of another run with samples blinded. **(B)** Receiver operating characteristic (ROC) curve plotting the true-positive rate against the false-positive rate along with a determination of the area under the curve (AUC). All diagnoses were confirmed at the time of death (n = 28).

Next, the 28 KASAR-processed SMG FFPE slide samples were blinded and plated to determine the reproducibility across RT-QuIC runs. The assay responses from those samples showed 93% of the sample runs (26 out of 28) were replicated based on the % Max of each sample on both RT-QuIC runs (Figure 3A). The *R* ^2^ = 0.92 shows that first-run results are an excellent predictor of second-run results, indicating that each sample is likely to give the same positive or negative result from one run to the next. Two PD samples failed to amplify on either run and although they are false negatives, it is known that not all PD patients display aggregates in the SMG. Thus, even these consistent negative results speak to the reliability of the assay. Of three ILBD SMG FFPE cases included in both the blinded and unblinded runs, none amplified. The receiver operating characteristic (ROC) curve generated from the run produced an area under the curve (AUC) of 0.775, which further supports the predictability of samples obtained from FFPE slides and processed with KASAR and is similar to published results for fresh frozen tissues (Figure 3B). In summary, after KASAR treatment, 11/13 PD SMG FFPE slide samples reacted consistently, while 2/13 were falsely negative. Of the negative samples, 0/19 reported as a false positive, and 19/19 reacted consistently across runs.

Collectively, our results demonstrate the applicability of the KASAR protocol across various sample types and forms. Postmortem samples from two different tissue types, brain and SMG, show that the protocol may be applied to a variety of tissue types. Three previously studied diseases, DLB, PD, and ILBD, as well as normal controls, were examined, and their RT-QuIC results were similar to those for fresh frozen tissues. Both DLB and PD samples resulted in amplification that was nearly indistinguishable from each other, and ILBD samples failed to react in RT-QuIC. Samples scraped from 1) a single slide, 2) a ribbon of serial cuts deposited directly in a tube from the microtome, and 3) blocks of FFPE tissue (e.g., core sample), were all successfully processed and analyzed.

## 4 Discussion

In this paper, we offer an efficient solution to utilizing FFPE samples for biomarker studies utilizing kinetic assays for amplification of biomarker protein aggregates. First, we explore the effects of well-established histopathological sample processing and storage on tissue seeding ability in the RT-QuIC assay. We then detail our KASAR protocol by which biomarker seed proteins from FFPE tissues can be recovered for analysis by RT-QuIC. Our results support the claim that KASAR is versatile and applicable across a variety of FFPE sample tissues for the recovery of kinetic assay seeding material. Using our simple single-tube recovery process, repeatable results for three different neurodegenerative diseases were obtained from ultra-low amounts of preserved tissue, representing source material that remains in a stable state at room temperature. The results also indicate that formalin fixation significantly reduces a sample’s ability to act as a seed in a kinetic assay using previously published sample processing protocols. Once a sample has been formalin-fixed, further processing *via* alcohol dehydration and paraffin embedding does not further impair a sample’s ability to seed a kinetic assay.

With neurodegenerative diseases, the ability to sample the most notably affected organ, the brain, prior to death is limited. Recent studies indicate more accessible tissues may also contain biomarkers earlier in the disease process (Manne et al., 2019; Manne et al., 2020). Because of the ease of storage and possible benefits to research, many hospitals and pathologists maintain extensive repositories of FFPE tissues. Although these samples were often originally submitted for screening or diagnosis of specific symptomologies or disorders, the potential remains for biomarkers of other diseases to be discovered in these archived samples as well. Along with the wealth of associated patient histories available, these repositories represent a huge source of potential data to be explored with protein aggregation kinetic assays.

Currently, many diseases are not detectable in tissue samples with traditional methods such as IHC or ELISA due to the difficultly in sampling disease tissue antemortem as well as the need for highly sensitive testing methods. Ultra-sensitive detection by protein aggregation kinetic assay can reveal the presence of biomarkers in samples long before the patient may experience classic symptoms. There is currently only one disease that can be clinically diagnosed by protein aggregation assay, Creutzfeldt-Jakob disease (CJD). In research, several other methods of detecting aggregated protein fibrils are available such as Western blotting, IHC, and ELISA testing. IHC is currently the only other detection method able to utilize formalin-fixed specimens for detection and analysis of aggregated protein fibrils.

Fenyi et al. (2021) recently indicated that formalin fixation could lead to false positive results in PMCA. Our RT-QuIC data presented here comparing fixed tissues cut from the same section as the fresh frozen specimen did not generate any false positives. The PMCA assay, like RT-QuIC, is a very sensitive assay that may be able to detect biomarker proteins prior to clinical symptoms indicative of disease. Given the natural tendency for these substrates to randomly aggregate, false-positive results could stem from early-stage synucleinopathy being detected in normal controls, or that the alternative fixation process is indeed responsible for the false positive results in the Fenyi et al. study.

Fenyi et al. also suggested that formalin fixation did not prevent fibrils from seeding an assay based on formalin treatment of pre-formed fibrils (PFFs) from pure protein. However, it is not clear if they used a sufficient amount of formalin. Since chemical reactions are heavily dependent upon the concentration of their reactants as well as environmental conditions (e.g., temperature, pH), it is possible the dilute formaldehyde added to the dilute pure protein mixed with absorbent buffer simply did not offer a realistic comparison to intact tissues where a fixed sample fibril would be found. Finally, another interesting observation from the Fenyi et al. study involves two unique locations of PD brain tissues. In each location of the 12 PD patients who were tested, 50% of the results were false negatives that failed to elicit a ThT response in the PMCA assay. This is very similar to the results obtained in the experiments presented here where fixed brain tissues also produced false negatives. Other sample locations were able to give more consistently true positive results and the results per patient were overall representative of the disease status, but in evaluating only the brain samples, the results, if not conclusions, are similar.

The loss of seeding ability after formalin fixation is likely due to formalin’s (CH2O) highly reactive nature and the bonds formed within the preserved tissues. It is hypothesized that recovering a sample’s seeding ability after heating in a tris solution occurs because tris acts as a scavenger molecule of the formalin-derived reactive compounds (Thacker et al., 2021). Through mechanisms that are still unclear, this process recovers the seeding ability of aggregated protein fibrils of the formalin-fixed tissue necessary to react in RT-QuIC.

Many protein aggregate structures are rich in hydrophobic beta-sheet structures, stacking one upon the other and forming distinctive fibrils (Sternbach et al., 1997). They can tolerate the harsh conditions of the KASAR protocol, such as heating well above body temperature for an extended period, without permanently losing their fibril structure. Indeed, the high-temperature tolerance of aSyn fibrils likely explains the KASAR protocol’s success with this particular species of protein aggregate. Brief exposure to high temperature (90°C) appears necessary for reaching the threshold to break the initial formalin-derived bond from the cellular proteins. Incubation at a slightly lower temperature (60°C) is thought to allow the formalin-freeing reactions to continue (Kawashima et al., 2014). Despite the extended incubations above normal body temperatures, aSyn is not degraded and either retains or regains its misfolded, aggregated conformation that effectively seeds protein aggregation kinetic assays.

Sample sonication is also necessary to restore kinetic assay seeding ability. Prior to sonication, the heat-treated samples appear mostly clear with some slight cloudiness or small particles visible. However, after sonication, almost all samples become visibly turbid. This degree of cloudiness does not occur in samples that are not heat-treated in tris buffer such as samples simply homogenized and sonicated in PBS. For heat-treated samples, those run through RT-QuIC without sonication exhibited weaker seeding activity than the same samples run after sonication (data not shown).

During this series of experiments, several sample-handling refinements occurred. Initially, samples were deparaffinized on the slide according to standard IHC protocols. Recovery of the majority of large tissue sections (≥1 cm^2^) was achievable with some difficulty. Particularly, the static charge effects of handling drying tissue flakes near the sample collection tube presented a significant challenge. Recovery of smaller samples, such as needle biopsy samples, proved to be nearly impossible due to static even when using the Haug U-ionizer. Even if introduced to the tube successfully, samples were so light in comparison to the tube as to be undetectable by highly sensitive scales, unless wetted with PBS or another buffer but the application and rate of evaporation confounded efforts to accurately weigh the tissue.

Efforts to resuspend samples in a specific volume of buffer, as determined by total surface area, while on the slide was slightly less problematic even though significant amounts of the minute samples clung to the sidewalls of even the highest quality low-binding pipettes. Alternatively, we warmed slides prior to deparaffinization so the paraffin and tissue sample could be scraped into the tube where the deparaffinization was done in a single-tube process. Warming was later replaced by the antistatic step of adding a small amount of highly evaporative liquid, such as 70%–100% ethanol, to the somewhat flaky samples. Xylene, in contrast, proved unsuitable for that step as it dissolves the paraffin and creates a sticky situation preventing sample transfer to the collection tube.

The most successful method used in this study was cutting numerous sections from the tissue block on the microtome and placing them directly into the sample tube for further processing. The cutting temperature of the block is optimized to produce a smooth ribbon of serial cuts that are easily gathered into a compact bouquet to be placed in the sample tube. Standard sections (e.g., 4–5 µm) are preferable as they result in rapid homogenization and efficient reactions without the need for special homogenization equipment.

**FIGURE 4:**
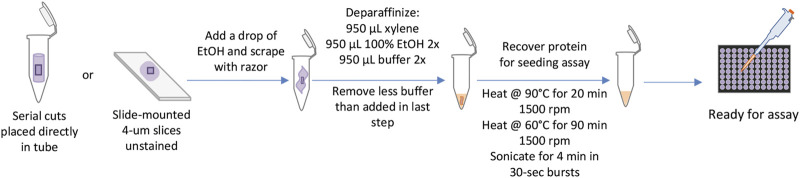
KASAR workflow. The workflow for processing FFPE samples or slides, from left to right. Starting with placing either serial slices cut from the block of embedded tissue directly into the tube or slide-mounted slices scraped from the glass with the addition of a small drop of ethanol. Next, the sample is deparaffinized in a single-tube process, then TSE buffer is added. The KASAR process is a combination of heating and mixing in the TSE buffer. The sample is then ready for downstream processes.

Before undergoing the KASAR protocol, formalin-fixed slide-mounted specimens can be stored at room temperature in slide cases, while unmounted ribbons of tissue sections can be stored on the shelf in 1.5-ml tubes. Samples can be left to air-dry overnight after the final step of deparaffinization but access to a SpeedVac allows samples to be dried more rapidly. During sample storage, the first volume of the buffer can be added to the deparaffinized sample and stored at -20°C until the remaining KASAR process resumes. After heating and sonication with the KASAR protocol, samples are either utilized immediately for RT-QuIC or stored at -80°C until needed (Figure 4).

The KASAR protocol was modeled after several other similar protocols being used in other disciplines while, most notably, closely monitoring the effects of harsh conditions on the final protein seeding ability. Despite several common laboratory events such as machine errors, extended incubations mid-process, changes in the manufacturer of reagents, regularly preparing fresh batch-specific reagents, and completely reprocessing samples for additional experiments, the protocol proved to be robust in giving repeatable results.

## Data Availability

The original contributions presented in the study are included in the article/supplementary material, further inquiries can be directed to the corresponding author.

## References

[B1] Al-Attas A. Assidi M. Al-Maghrabi J. Dallol A. Schulten H. J. Abu-Elmagd M. (2016). Enhancement of pathologist's routine practice: Reuse of DNA extracted from immunostained formalin-fixed paraffin-embedded (FFPE) slides in downstream molecular analysis of cancer. Cancer genomics & proteomics 13, 399–406. 27566658 PMC5070629

[B2] Berrino E. Annaratone L. Miglio U. Maldi E. Piccinelli C. Peano E. (2020). Cold formalin fixation guarantees DNA integrity in formalin fixed paraffin embedded tissues: Premises for a better quality of diagnostic and experimental Pathology with a specific impact on breast cancer. Front. Oncol. 10, 173. 10.3389/fonc.2020.00173 32140450 PMC7042205

[B3] Coscia F. Doll S. Bech J. M. Schweizer L. Mund A. Lengyel E. (2020). A streamlined mass spectrometry-based proteomics workflow for large-scale FFPE tissue analysis. J. pathology 251, 100–112. 10.1002/path.5420 32154592

[B4] Eltoum I. Fredenburgh J. Myers R. B. Grizzle W. E. (2001). Introduction to the theory and practice of fixation of tissues. J. Histotechnology 24, 173–190. 10.1179/his.2001.24.3.173

[B5] Fenyi A. Duyckaerts C. Bousset L. Braak H. Del Tredici K. Melki R. (2021). Seeding propensity and characteristics of pathogenic αSyn assemblies in formalin-fixed human tissue from the enteric nervous system, olfactory bulb, and brainstem in cases staged for Parkinson's disease. Cells 10, 139. 10.3390/cells10010139 33445653 PMC7828121

[B6] Frazer Z. Yoo C. Sroya M. Bellora C. DeWitt B. L. Sanchez I. (2020). Effect of different proteinase K digest protocols and deparaffinization methods on yield and integrity of DNA extracted from formalin-fixed, paraffin-embedded tissue. J. Histochem Cytochem 68, 171–184. 10.1369/0022155420906234 32043912 PMC7045302

[B7] García-Vence M. Chantada-Vazquez M. D. P. Sosa-Fajardo A. Agra R. Barcia de la Iglesia A. Otero-Glez A. (2021). Protein extraction from FFPE kidney tissue samples: A Review of the literature and characterization of techniques. Front. Med. 8, 657313. 10.3389/fmed.2021.657313 PMC8158658 34055835

[B8] Griesser E. Wyatt H. Ten Have S. Stierstorfer B. Lenter M. Lamond A. I. (2020). Quantitative profiling of the human substantia nigra proteome from laser-capture microdissected FFPE tissue. Mol. Cell. proteomics MCP 19, 839–851. 10.1074/mcp.RA119.001889 32132230 PMC7196589

[B9] Kapoor S. Lu G. van den Berg N. S. Krishnan G. Pei J. Zhou Q. (2021). Effect of formalin fixation for near-infrared fluorescence imaging with an antibody-dye conjugate in head and neck cancer patients. Mol. imaging Biol. 23, 270–276. 10.1007/s11307-020-01553-1 33078373

[B10] Kawashima Y. Kodera Y. Singh A. Matsumoto M. Matsumoto H. (2014). Efficient extraction of proteins from formalin-fixed paraffin-embedded tissues requires higher concentration of tris(hydroxymethyl)aminomethane. Clin. proteomics 11, 4. 10.1186/1559-0275-11-4 24484752 PMC3922997

[B11] Lai Z. W. Weisser J. Nilse L. Costa F. Keller E. Tholen M. (2016). Formalin-fixed, paraffin-embedded tissues (FFPE) as a robust source for the profiling of native and protease-generated protein amino termini. Mol. Cell. proteomics MCP 15, 2203–2213. 10.1074/mcp.O115.056515 27087653 PMC5083106

[B12] Manne S. Kondru N. Hepker M. Jin H. Anantharam V. Lewis M. (2019). Ultrasensitive detection of aggregated α-synuclein in glial cells, human cerebrospinal fluid, and brain tissue using the RT-QuIC assay: New high-throughput neuroimmune biomarker assay for parkinsonian disorders. J. Neuroimmune Pharmacol. 14, 423–435. 10.1007/s11481-019-09835-4 30706414 PMC6669119

[B13] Manne S. Kondru N. Jin H. Anantharam V. Huang X. Kanthasamy A. (2020). α-Synuclein real-time quaking-induced conversion in the submandibular glands of Parkinson's disease patients. Mov. Disord. 35, 268–278. 10.1002/mds.27907 31758740 PMC7102508

[B14] Mason J. T. (2016). Proteomic analysis of FFPE tissue: Barriers to clinical impact. Expert Rev. proteomics 13, 801–803. 10.1080/14789450.2016.1221346 27491521

[B15] Nguyen H. T. Tatipamula V. B. Do D. N. Huynh T. C. Dang M. K. (2022). Retrieving high-quality genomic DNA from formalin-fixed paraffin-embedded tissues for multiple molecular analyses. Prep. Biochem. Biotechnol. 52, 48–55. 10.1080/10826068.2021.1923030 34047684

[B16] Panchal N. K. Bhale A. Chowdary R. Verma V. K. Beevi S. S. (2020). PCR amplifiable DNA from breast disease FFPE section for mutational analysis. J. Biomol. Tech. JBT 31, 1–6. 10.7171/jbt.20-3101-001 31695579 PMC6822607

[B17] Pöschel A. Beebe E. Kunz L. Amini P. Guscetti F. Malbon A. (2021). Identification of disease-promoting stromal components by comparative proteomic and transcriptomic profiling of canine mammary tumors using laser-capture microdissected FFPE tissue. Neoplasia (New York, N.Y.) 23, 400–412. 10.1016/j.neo.2021.03.001 33794398 PMC8042244

[B18] Rourke M. B. Padula M. P. (2016). Analysis of formalin-fixed, paraffin-embedded (FFPE) tissue via proteomic techniques and misconceptions of antigen retrieval. BioTechniques 60, 229–238. 10.2144/000114414 27177815

[B19] Sternbach G. Dibble C. L. Varon J. (1997). From Creutzfeldt-Jakob disease to the mad cow epidemic. J. Emerg. Med. 15, 701–705. 10.1016/s0736-4679(97)00152-2 9348063

[B20] Thacker J. S. Andersen D. Liang S. Zieniewicz N. Trivino-Paredes J. S. Nahirney P. C. (2021). Unlocking the brain: A new method for western blot protein detection from fixed brain tissue. J. Neurosci. Methods 348, 108995. 10.1016/j.jneumeth.2020.108995 33202258

